# Methodological considerations for combining wastewater-based epidemiology with survey research

**DOI:** 10.1186/2049-3258-73-S1-P29

**Published:** 2015-09-17

**Authors:** Janelle Van Wel, Juliet Kinyua, Alexander Van Nuijs, Guido Van Hal, Adrian Covaci

**Affiliations:** 1 Antwerp University, Wilrijk, Antwerp, Belgium

## 

Population surveys are the most used technique to make estimations on drug use, but suffer from report biases. Wastewater-based epidemiology (WBE) collects wastewater samples and analyses them for the presence of drugs and their metabolites. This novel technique has not yet been compared to the results of more conventional epidemiological techniques, which was the goal of the current study. A website was opened during a 12-week period (autumn 2014) on which inhabitants of a selected community (N=29,083) were asked to indicate their drug use in the past week. Concomitant wastewater samples were taken from the wastewater treatment plant (WWTP) collecting from the community.

Results from both the survey and wastewater study matched national and international trends, but caution should be exercised in combining the two approaches. Low response rates on the survey (average N=263 weekly, 1% of the population) illustrate the dependency on the willingness of inhabitants to participate. It is not clear why response rates were so low. Using a mixed method might be more reliable (e.g. apply random sampling for the general population and use a different technique for illicit drug users), but has its own disadvantages. Data on drug consumption using WBE was shown to be relatively easy to obtain and the results were reliable. Large events are picked up in wastewater data and significant differences occurred over sampling weeks; thus, there is an increased chance of error in the data if only 1 week is used to draw conclusions on drug use in the population.

Future research on combining the two approaches should focus on either a more general approach, e.g. national population surveys, or take place in a more focused setting, such as festivals, where a higher degree of drug use can be expected. Also, more attention should be paid to selecting appropriate sampling periods.

